# Dynamics of Autotrophic Marine Planktonic *Thaumarchaeota* in the East China Sea

**DOI:** 10.1371/journal.pone.0061087

**Published:** 2013-04-02

**Authors:** Anyi Hu, Zao Yang, Chang-Ping Yu, Nianzhi Jiao

**Affiliations:** 1 Key Laboratory of Urban Environment and Health, Institute of Urban Environment, Chinese Academy of Sciences, Xiamen, China; 2 State Key Laboratory of Marine Environmental Science, Xiamen University, Xiamen, China; University of Delaware, United States of America

## Abstract

The ubiquitous and abundant distribution of ammonia-oxidizing *Thaumarchaeota* in marine environments is now well documented, and their crucial role in the global nitrogen cycle has been highlighted. However, the potential contribution of *Thaumarchaeota* in the carbon cycle remains poorly understood. Here we present for the first time a seasonal investigation on the shelf region (bathymetry≤200 m) of the East China Sea (ECS) involving analysis of both thaumarchaeal 16S rRNA and autotrophy-related genes (acetyl-CoA carboxylase gene, *accA*). Quantitative PCR results clearly showed a higher abundance of thaumarchaeal 16S and *accA* genes in late-autumn (November) than summer (August), whereas the diversity and community structure of autotrophic *Thaumarchaeota* showed no statistically significant difference between different seasons as revealed by thaumarchaeal *accA* gene clone libraries. Phylogenetic analysis indicated that shallow ecotypes dominated the autotrophic *Thaumarchaeota* in the ECS shelf (86.3% of total sequences), while a novel non-marine thaumarchaeal *accA* lineage was identified in the Changjiang estuary in summer (when freshwater plumes become larger) but not in autumn, implying that Changjiang freshwater discharge played a certain role in transporting terrestrial microorganisms to the ECS. Multivariate statistical analysis indicated that the biogeography of the autotrophic *Thaumarchaeota* in the shelf water of the ECS was influenced by complex hydrographic conditions. However, an in silico comparative analysis suggested that the diversity and abundance of the autotrophic *Thaumarchaeota* might be biased by the ‘universal’ thaumarchaeal *accA* gene primers Cren529F/Cren981R since this primer set is likely to miss some members within particular phylogenetic groups. Collectively, this study improved our understanding of the biogeographic patterns of the autotrophic *Thaumarchaeota* in temperate coastal waters, and suggested that new *accA* primers with improved coverage and sensitivity across phylogenetic groups are needed to gain a more thorough understanding of the role of the autotrophic *Thaumarchaeota* in the global carbon cycle.

## Introduction

The Archaea, one of the three domain systems, was introduced in 1977 by Carl Woese based on genes encoding the small ribosomal subunit (namely, 16S rRNA gene) [1]. Members of the Archaea were originally thought to be restricted to extreme environments, but are now known to be very common in marine environments and elsewhere [2]. The marine planktonic Archaea consist of both mesophilic *Euryarchaeota* and *Crenarchaeota*, the latter dominating planktonic archaeal communities [3], [4], and recently proposed as a novel archaeal phylum, the *Thaumarchaeota* [5], [6]. There is growing evidence that members of the *Thaumarchaeota* play an important role in carbon and nitrogen biogeochemical cycling in both marine and terrestrial environments [7]–[10].

Previous metagenomic investigations of marine and soil microbial communities have revealed that the *Thaumarchaeota* may be involved in ammonia oxidation, an essential microbially mediated process for converting reduced N into oxygenated N [11], [12]. Further studies on the enrichment and isolation of several thaumarchaeal cultures provide strong evidence that the *Thaumarchaeota* have the capability to oxidize ammonia to nitrite [13]–[15]. Quantitative analyses of *amoA* genes showed that ammonia-oxidizing Archaea (AOA) are ubiquitous in natural and man-made environments, and outnumber their bacterial counterparts in many environments, suggesting that the *Thaumarchaeota* play an essential role in global nitrification [16]. On the other hand, the autotrophic carbon metabolism of the *Thaumarchaeota* has received increasing attention with recognition of a their significant role in marine carbon cycling as revealed by both genomic and physiological evidence [3], [17]–[18].

The 3-hydroxypropionate/4-hydroxybutyrate (3-HP/4-HB) CO_2_ fixation pathway was first found in a thermophilic crenarchaeon *Metallospharea sedula* by Berg *et al*. (2007) [19] but it is now known as a unique CO_2_ fixation pathway for the *Thaumarchaeota* and found in all available thaumarchaeal genomes [17], [18], [20]–[23]. The *accA* gene, encoding the α-subunit of acetyl-CoA carboxylase, one of the conversed enzymes in this 3-HP/4-HB pathway proves to be a useful molecular marker for the autotrophic *Thaumarchaeota* in the environment [7], [24], [25]. Recent open ocean studies show that the ratios of thaumarchaeal *accA* genes to 16S rRNA or *amoA* genes are lower than 1∶1 in the euphotic zone but increase with depth, implying that the marine planktonic *Thaumarchaeota* may grow heterotrophically in the upper water of the open ocean [7], [26]. However, until now no investigation has examined the distribution of the autotrophic *Thaumarchaeota* or how this functional group responds to environmental gradients in the coastal ocean.

The East China Sea (ECS), located in the North West Pacific, is the largest continental shelf sea in the temperate zone. The hydrological conditions in the ECS are extremely complicated and dynamic, due to the interactions between the nutrient enriched freshwater from the Changjiang (Yangtze River) and the oligotrophic oceanic water of the Kuroshio Current [27], [28]. It is thus an ideal ecosystem for ecological studies of microbial dynamics along both temporal and spatial dimensions. A study using clone libraries of 16S rRNA genes shows that the marine Group 1.1a *Thaumarchaeota* (Group 1.1a) is prevalent in the estuarine region of the ECS [29], while another work suggests that the community structure of sediment AOA in the Changjiang estuary and the adjacent ECS shifts along hydrological gradients [30]. In a recent investigation, we report clear niche partitioning of Group 1.1a within the water column of the open region of the ECS [26]. However, the temporal and spatial variations of the marine planktonic *Thaumarchaeota* in the ECS shelf are still poorly understood, especially in the case of the autotrophic functional group. In the present study, we compared the abundance, distribution and community structure of the autotrophic *Thaumarchaeota* in the shelf waters (bathymetry≤200 m) of the ECS between summer and autumn using clone libraries and quantitative PCR (qPCR) of thaumarchaeal 16S rRNA and *accA* genes.

## Materials and Methods

### Sampling

**Figure 1:**
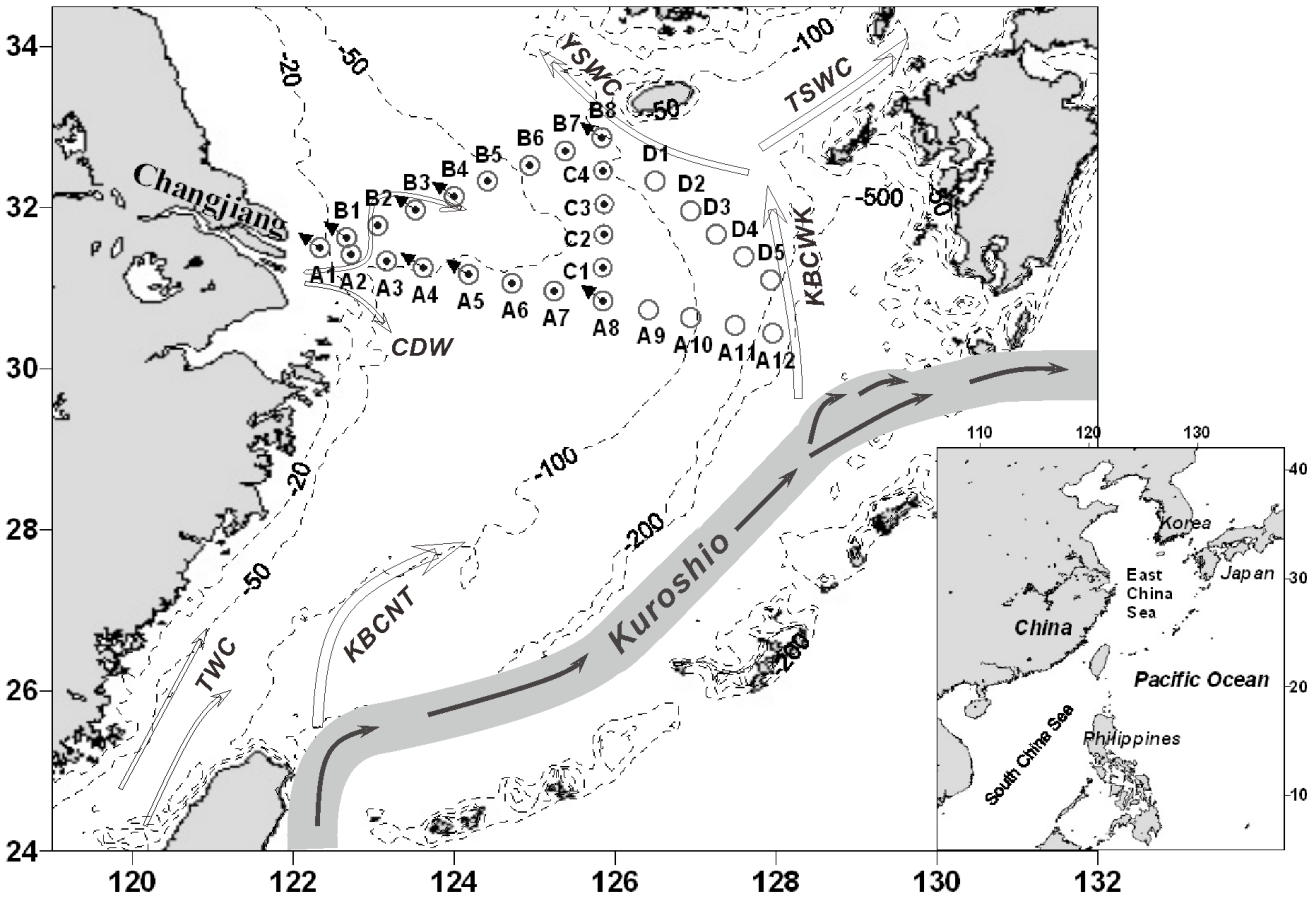
Map of the study region and location of the field stations where surface water samples were collected in autumn 2007 and summer 2008 (open circle with dot in center); where surface water samples were only collected in autumn 2007 (open circle); and stations where depth profiles samples were collected in two autumn stations (B3 and B8) and seven summer stations (B1, B4, B8, A1, A4, A5 and A8). CDW: Changjiang Diluted Water; TWC: Taiwan Warm Current; KBCNT: Kuroshio Branch Current north of Taiwan; KBCWK: Kuroshio Branch Current West of Kyushu; TSWC: Tsushima Strait Warm Current; YSWC: Yellow Sea Warm Current; Kuroshio: Kuroshio Current. This figure was adapted and updated from Figure 1 in reference 26.

Sampling was conducted in the late autumn (3^rd^ and 24^th^ November 2007) and summer (7^th^ and 18^th^ August 2008) on board the R/V ‘Dongfanghong #2’ at 20 and 29 stations, respectively, along four cross-shelf transects (transect A, B, C and D) from the Changjiang estuary to the Kuroshio area (Fig. 1). No specific permits were required for these field studies in that: a) no specific permission was required for these locations/activities; b) the locations were not privately-owned or protected in any way; and c) the field studies did not involve endangered or protected species.

Water samples were collected with 12 L Niskin bottles mounted in a SeaBird CTD (conductivity-temperature-depth) system. Water samples (0.5–2L) were pre-filtered through 20 µm mesh (Millipore, Billerica, MA, USA) and subsequently filtered onto 0.2-μm pore-size polycarbonate filters (Millipore) at a pressure of <0.03 MP. The filters were immediately frozen and stored at −80°C until further analysis.

### Nucleic acid extraction

The microbial community genomic DNA was extracted using the UltraClean Soil DNA kit (MoBio, San Diego, CA, USA), as processed in reference 7. DNA integrity and size was checked in a 0.8% agarose gel stained with SYBR Green I (Molecular Probes, Eugene, OR, USA), and the concentrations were quantified in duplicate using FlexStation*®* 3 (Molecular Devices, Sunnyvale, CA, USA) with a Quant-iT™ dsDNA HS Assay Kit (Molecular Probes). A standard curve was generated using known amounts of Lambda DNA (Molecular Probes).

### Quantitative PCR analysis

The abundance of thaumarchaeal 16S rRNA and *accA* genes were quantified in triplicate with an ABI PRISM 7500 system (Applied Biosystems, Foster City, CA, USA) using the SYBR Green based method. Thaumarchaeal 16S rRNA gene copy numbers were determined using primers GI_751F and GI_956R [31] and the following reaction chemistry was used: a 20 µL reaction mixture consisting of 10 µL of SYBR GreenER™-qPCR SuperMix Universal (Molecular Probes), 50 nM ROX dye, 5 µg BSA, plus 0.4 µM of each primer and 1 µL of template (1–10 ng) was used. For quantification of the abundance of thaumarchaeal *accA* genes, the following reaction mixture was used: 10 µL of SYBR*®* Premix Ex Taq™ (TakaRa, Dalian, China), 50 nM ROX dye, 5 µg BSA, 0.4 µM primers (Cren529F/Cren981R) [24] and 1 µL template DNA of 1–10 g in a final volume of 20 µL. The thermal cycling conditions were the same as those used in our previous study [7]. The specificity of qPCR reactions was confirmed using melting curve analysis and agarose gel electrophoresis after amplification.

The plasmids used as standards in qPCR were constructed previously [7]. The concentrations of plasmid DNAs were determined using a Quant-iT™ dsDNA BR Assay Kit (Molecular Probes). Ten-fold serial dilutions of a known number of plasmids were subjected to qPCR assay in triplicate to generate an external standard curve. The assay efficiency of the thaumarchaeal 16S rRNA gene was 102–108% with R^2^ values more than 0.997, while the corresponding values for the thaumarchaeal *accA* gene were 90–98% and 0.998.

### PCR amplification of thaumarchaeal *accA* genes and clone library analyses

The thaumarchaeal *accA* gene was amplified with primers Cren529F/Cren981R [24], but the nested PCR strategy was employed when few positive PCR products were obtained from the summer samples. The PCR mixture (30 µL) contained 15 µL FailSafe Premix F (Epicentre Biotechnologies, Madison, WI, U.S.A.), 0.5 µM of each primer, 1 U of Ex Taq DNA polymerase (TakaRa), 6 µg BSA and 1 µL (c. 3–20 ng DNA) of template. The PCRs were run for 35 cycles, following the PCR conditions described in the literature listed previously. Three independent PCR products were pooled and purified with an Agarose Gel DNA Purification kit (TaKaRa), ligated into the pMD18-T vector (TaKaRa) and then transformed into competent *Escherichia coli* DH5α (TaKaRa). Positive clones were screened using PCR re-amplification with vector primers M-13F/M-13R and randomly selected for sequencing with an ABI 3730 XL sequencer (Applied Biosystems).

### Phylogenetic analysis

The thaumarchaeal *accA* gene sequences, along with their closest relatives retrieved from GenBank, were imported into ARB [32]. The sequences were first translated and aligned using Clustal W in ARB, and then the nucleotides were realigned according to their protein alignment. Ambiguously and incorrectly aligned positions were corrected manually using the ARB-edit tool. A sequence bases frequency filter was created to remove ambiguous positions and columns containing gaps. The maximum likelihood (ML) tree was generated using RAxML 7.2.8 [33] at the CIPRES website (http://www.phylo.org), and a general time-reversible model plus gamma distribution plus the invariant sites model of molecular evolution. Tree topologies were evaluated based on 1,000 bootstrap replicates.

### Diversity indices and statistical analyses

The thaumarchaeal *accA* gene sequences were grouped into operational taxonomic units (OTUs) based on a 5% sequences divergence cutoff using MOTHUR v1.22.0 with the furthest neighbor algorithm [34]. Diversity indices including the nonparametric richness estimator Chao1, the Shannon diversity (H′) and the sampling Coverage index (C) were also calculated using MOTHUR.

Community classification of different thaumarchaeal *accA* gene clone libraries was compared using non-phylogenetic (OTU-based) clustering and phylogenetic (weighted UniFrac) clustering analyses [35]. Analysis of similarities (ANOSIM) was used to test the significance of the autotrophic thaumarchaeal community structures between seasons. A Mantel test was performed to evaluate whether there was a correlation between autotrophic thaumarchaeal communities and environmental variables or geographic distance. Correlations between thaumarchaeal gene abundances and environmental variables were calculated using non-parametric Spearman's correlation since normality of distribution of the individual data sets was not always met. Analyses were conducted with the PAST v1.92 program [36].

### Nucleotide sequence accession number

The non-redundant sequences reported in this study have been deposited in the GenBank database under accession numbers JQ952676 to JQ952734.

## Results

### Environmental parameters

**Figure 2:**
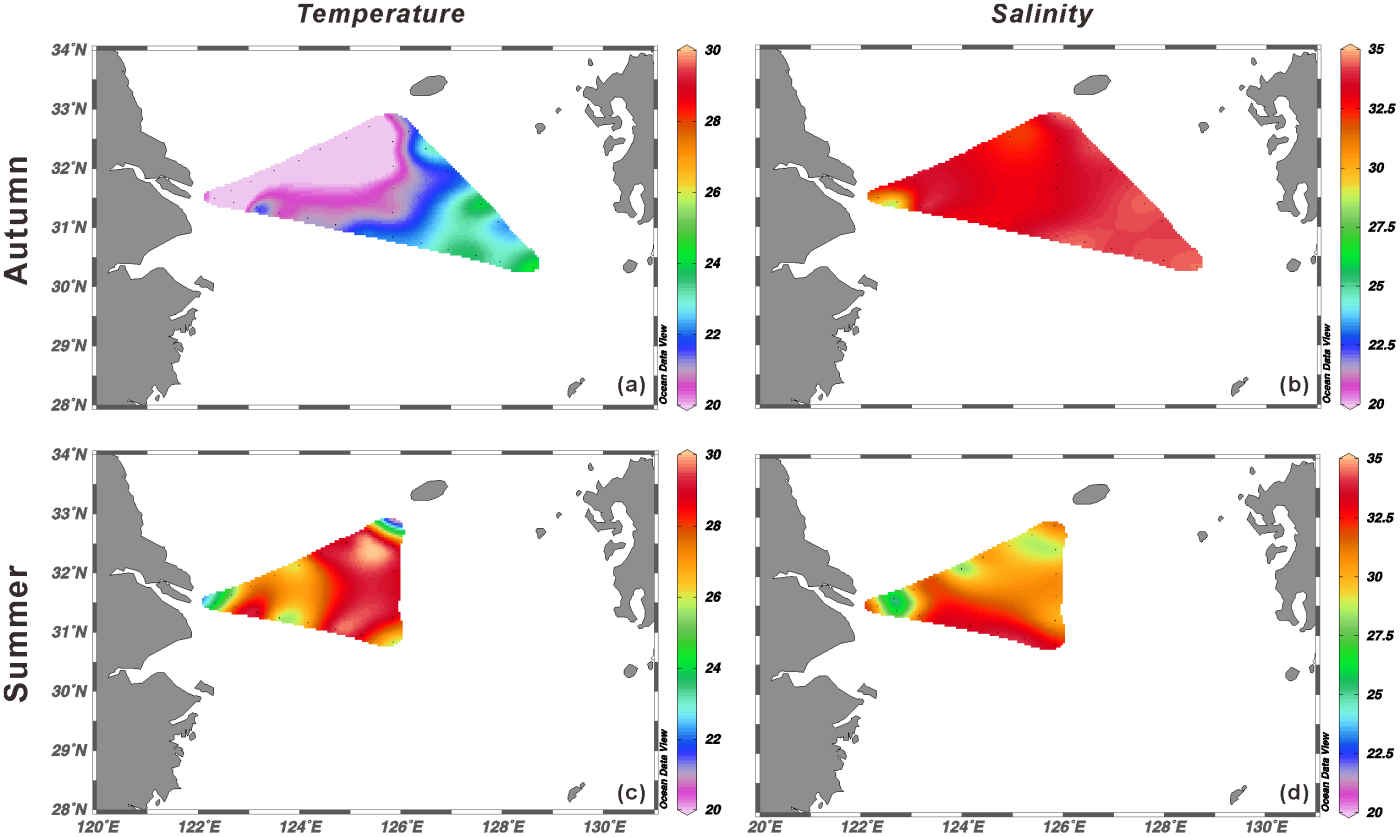
Surface profiles of the temperature (a, c) and salinity (b, d) in autumn and summer in the shelf area of the ECS.

**Figure 3:**
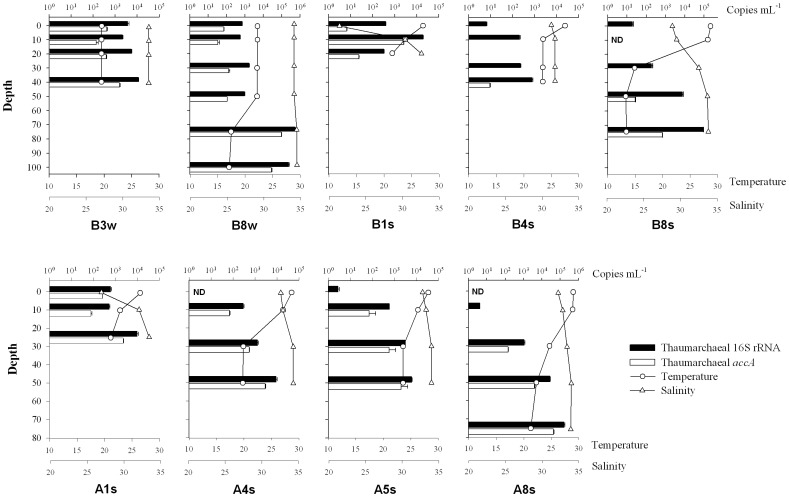
Depth profiles of temperature, salinity, and the abundance of thaumarchaeal 16S rRNA and *accA* genes in two autumn stations (B3w and B8w) and seven summer stations (B1s, B4s, B8s, A1s, A4s, A5s and A8s). Bars denote one standard deviation of the triplicate qPCR determination.

The Kuroshio Current along the ECS shelf edge originates from the West Pacific Warm Pool, and is characterized by high temperature and high salinity. Conversely, low salinity and low temperature content are identified from the coastal water [27], [37]. Because of the influences of different water masses, hydrographic conditions in the ECS are very different in autumn and summer (Figs 2 and 3). As seen in Fig. 2 (which was derived from in situ measurements of temperature and salinity), the cold freshwater plume from the Changjiang and the shoreward intrusion of the warm water were less extensive in autumn, while in summer greater shoreward intrusion of the offshore warm water could be observed. Also, the summer freshwater discharges from the Changjiang were more intense, and the majority of discharges intruded into transect B, although several high salinity water tongues were found in this transect due to the interactions among the Changjiang Diluted Water (CDW), oceanic water from the Kuroshio Current and Taiwan Warm Current (Fig. 2).

Two autumn and seven summer stations were chosen (as indicated in Fig. 1) to investigate the depth distribution of autotrophic *Thaumarchaeota*, and vertical profiles of the temperature and salinity at these stations are shown in Fig. 3. The salinity had constant values in the autumn stations since the CDW was weaker in autumn than in summer, but the thermocline was deeper in autumn than in summer (Fig. 3).

### Abundance of 16S rRNA and *accA* genes of the planktonic *Thaumarchaeota*

**Figure 4:**
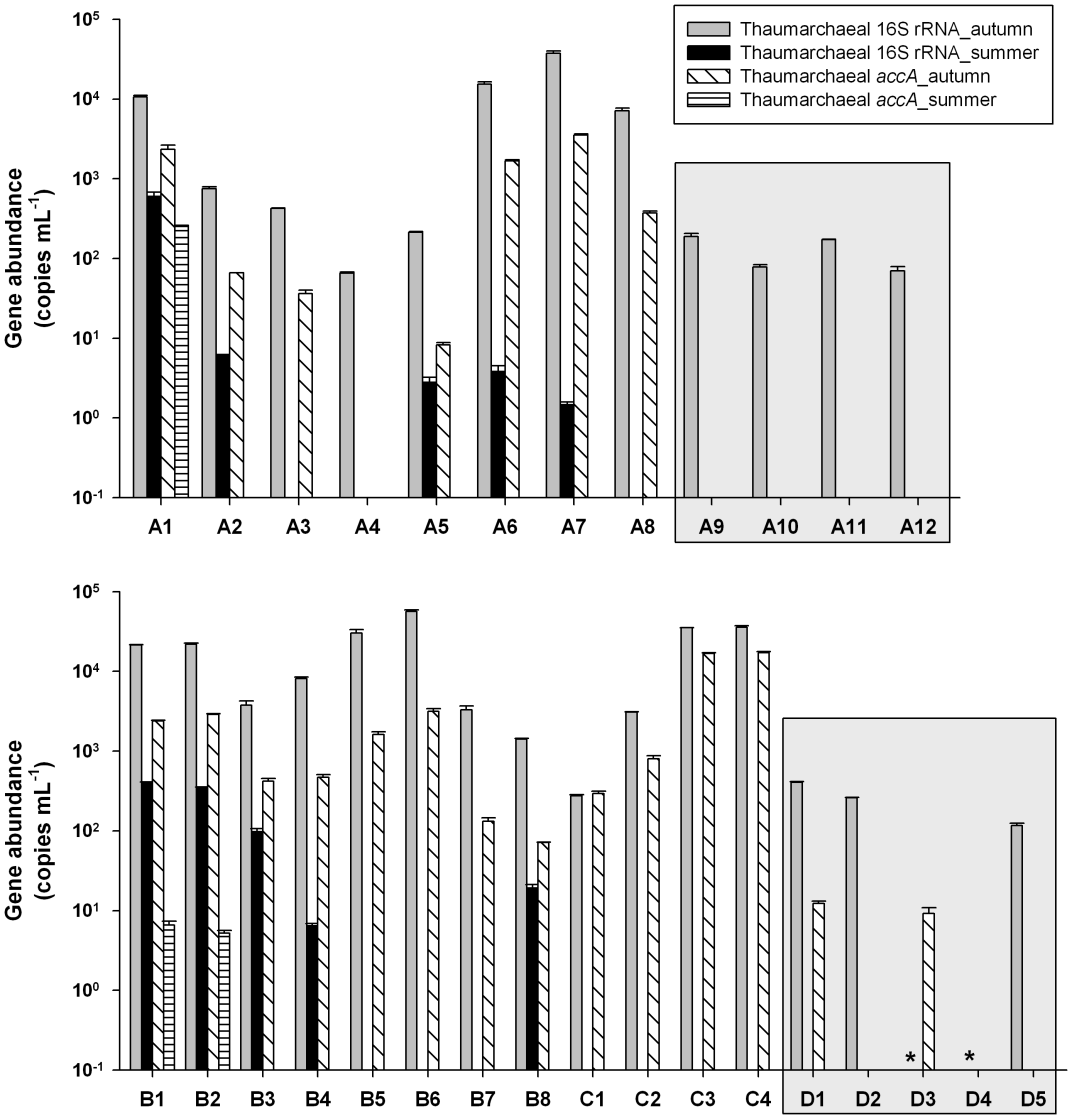
Surface distribution of the abundance of thaumarchaeal 16S rRNA and *accA* genes in the ECS. Bars denote one standard deviation of the triplicate qPCR determination. Asterisks indicate that abundance of thaumarchaeal 16S rRNA gene was not determined in stations D3 and D4 since there was not enough environmental DNA. Surface water samples were only collected in autumn of 2007 (grey shaded area).

qPCR results demonstrated that both thaumarchaeal 16S rRNA and *accA* genes were significantly more abundant during the autumn than the summer (Mann-Whitney, *P*<0.01) (Figs 3 and 4). Vertically, both thaumarchaeal 16S rRNA and *accA* genes were more abundant in subsurface water than those in surface water, with clear increases in gene abundance with depth (Fig. 3). In surface water, thaumarchaeal 16S rRNA gene abundance ranged from 6.50 × 10^4^ to 5.66 × 10^7^ copies L^−1^ in autumn, whereas only 10 of 19 summer samples could be detected with thaumarchaeal 16S rRNA genes, and their abundance varied from 1.46 × 10^3^ to 5.99 × 10^5^ copies L^−1^ (Fig. 4). The abundance of thaumarchaeal *accA* genes exhibited a similar trend to that of thaumarchaeal 16S rRNA gene abundance, ranging from below detection limit to 1.72 × 10^7^ copies L^−1^ in the ECS surface water (Fig. 4).

A linear regression analysis indicated that the thaumarchaeal 16S rRNA and *accA* genes were significantly correlated with each other (R^2^ = 0.87; *P*<0.001). However, the *accA* genes were almost always less abundant than the thaumarchaeal 16S rRNA genes, with an averaged relative proportion of 16.3% ± 2.6% of thaumarchaeal 16S rRNA genes. Correlation relationships between environmental parameters and thaumarchaeal 16S rRNA or *accA* gene abundances were also calculated. Collectively, only temperature showed a negative correlation with thaumarchaeal 16S rRNA (Spearman rank correlation, r = −0.65, *P*<0.001) and *accA* (r = −0.44, *P*<0.01) gene abundances. Within each season, thaumarchaeal 16S rRNA gene abundance exhibited significant correlation with temperature (autumn: r = −0.62, *P*<0.001; summer: r = −0.74, *P*<0.001) and salinity (autumn: r = −0.36, *P*<0.05; summer: r = −0.45, *P*<0.05). However, thaumarchaeal *accA* gene abundance was found to be strongly correlated with salinity in autumn (r = −0.64, *P*<0.01) but with temperature in summer (r = −0.60, *P*<0.001).

### Genetic diversity of thaumarchaeal *accA* genes

To explore the diversity and structure of the autotrophic thaumarchaeal community in the ECS, nine and 10 thaumarchaeal *accA* gene clone libraries were analyzed from autumn and summer samples, respectively (Table 1). A total of 620 thaumarchaeal *accA* gene sequences were obtained, and could be grouped into 59 OTUs at a 5% divergence cutoff value at the DNA level. The numbers of OTUs per sample varied between three and 13, and was highest in B8w_75 and A1s_0 and lowest in A7s_0 (Table 1). The values of the sampling Coverage (C) were generally higher (78.3%–100%) except for the surface water sample obtained from estuarine station A1 in summer (A1s_0), indicating that most clone libraries adequately covered the diversity of thaumarchaeal *accA* genes. Statistical analysis indicated that there was no significant difference in thaumarchaeal *accA* gene diversity between the autumn and summer samples (Mann-Whitney test, *P*>0.1) (Table 1).

**Table 1:** Diversity indices of thaumarchaeal *accA* clone libraries from the ECS.

Season	Samples	n	No. of OTUs	C (%)	H'	Chao1
Autumn	A1w_0	25	8	84.0	1.75	14
	A4w_0	26	8	88.5	1.62	9
	A8w_0	25	10	78.3	2.05	15
	A11w_0	34	6	97.1	1.50	6
	A12w_0	35	7	94.3	1.29	8
	B3w_0	33	6	91.0	1.38	9
	B3w_40	31	6	93.6	1.44	7
	B8w_0	36	11	83.3	1.79	16
	B8w_75	38	13	81.6	2.20	34
Summer	A1s_0	25	13	68.0	2.21	19
	A1s_25	38	7	92.1	1.12	8
	A5s_10	37	8	89.2	1.37	11
	A5s_50	39	11	82.1	1.54	18
	A7s_0	30	3	100	0.84	3
	A8s_75	39	9	89.7	1.45	11
	B4s_10	30	4	96.7	0.90	4
	B4s_50	35	8	91.4	1.61	10
	B8s_0	32	5	96.9	1.05	5
	B8s_75	34	10	88.2	1.94	13

**Figure 5:**
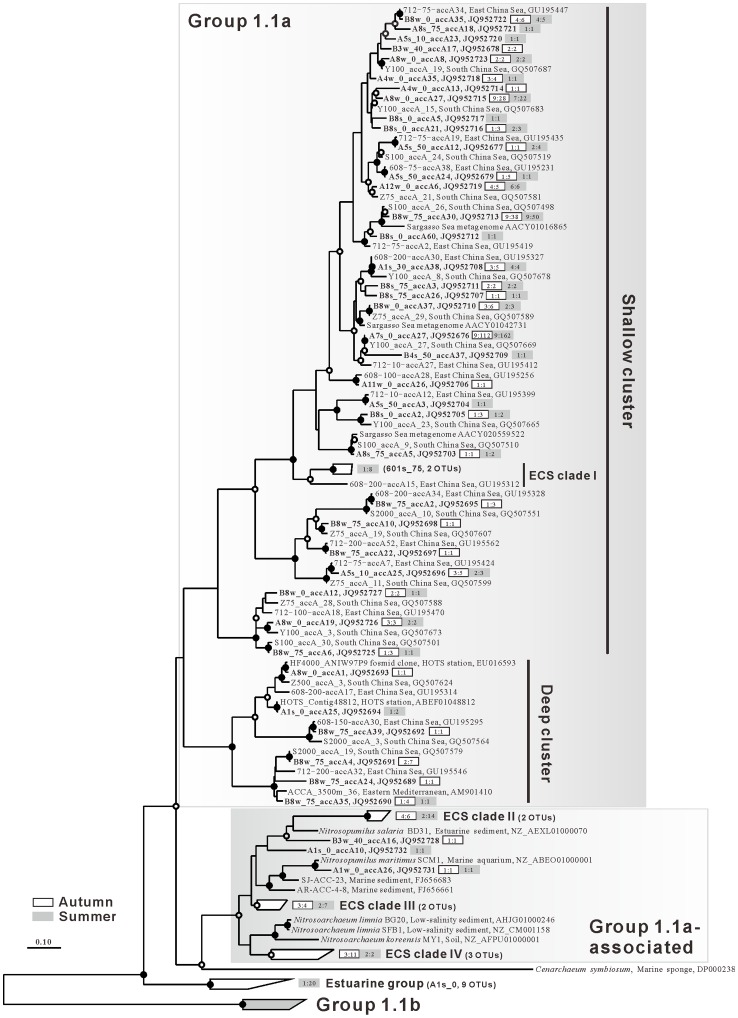
Maximum likelihood tree of thaumarchaeal *accA* gene sequences. *Bacteriodes fragilis* NCTC 9343 (CR626927) was used as the outgroup to root the tree. Sequences with≤5% distance cutoff are represented by only one of them. Clone sequences recovered in this study are in boldface. Boxes behind the representative sequences indicate samples obtained from autumn (white) or summer (grey). The number in the box indicates the number of occurrences and the number of clones in the OTUs found in that season. For example, OTU A7s_0_accA27 (JQ952676) was found in nine autumn stations with 112 sequences and nine summer stations with 162 sequences. Bootstrap values greater than 80% and 50% were shown, respectively, by solid and open circles in the nodes. Nodes without circles were not resolved. Scale bar indicates 0.1 nucleotide substitution per site.

Phylogenetic analysis indicated that the thaumarchaeal *accA* sequences recovered in this study fell into three major lineages: Group 1.1a, Group 1.1a-associated and Estuarine group (Fig. 5). The majority of the sequences (552 out of 620, 89%) fell into Group 1.1a and could be further divided into two subclusters (‘shallow’ and ‘deep’ clusters) which were previously proposed to represent depth-stratified ecotypes of pelagic *Thaumarchaeota* based on the *amoA* [38], [39] and *accA* genes [7]. The shallow cluster contained 535 *accA* gene sequences grouped with those from the open ocean waters of the ECS [26], the South China Sea (SCS) [7] and the Sargasso Sea [12] (Fig. 5). Several thaumarchaeal *accA* OTUs dominated this cluster. For instance, OTUs A8w_0_accA27, B8w_75_accA30 and A7s_0_accA27 accounted for 9.3% (50 out of 535), 16.4% (88 out of 535) and 51.2% (274 out of 535) of the sequences of the shallow cluster, respectively. These abundant autotrophic thaumarchaeal ‘shallow species’ were cosmopolitan in the ECS, i.e. they were found in most clone libraries (≥ 16) (Fig. 5). Consistent with the shallow depth characteristics of the sampling area, only a small fraction of all sequences (16 out of 620, 2.6%) were grouped phylogenetically with sequences from meso- or bathypelagic waters including the SCS [7], North Pacific Gyre [40] and Mediterranean Sea [24].

The remaining thaumarchaeal *accA* sequences fell exclusively into the Group 1.1a-associated lineage in which the environmental sequences retrieved from coastal sediment [41], and all available cultured thaumarchaeons (as at 30 April 2012) including *Nitrosopumilus salaria* [20], *Nitrosopumilus maritimus* [15], *Nitrosoarchaeum limnia* [22], [23] and *Nitrosoarchaeum koreensis* [21] were affiliated with this lineage.

Most *accA* gene sequences from the summer estuarine sample A1s_0 (20 out of 25) formed a monophylogenetic lineage distantly related to Group 1.1b with no environmental sequences clustering together (Fig. 5). ML bootstrap analyses indicated that this phylogenetic node was supported by values >80% (Fig. 5). Therefore, we provisionally named it here as the Estuarine group, with any two sequences in this cluster sharing 83–94% nucleic acid identity and 94–99% amino acid identity. Indeed, these sequences were distantly related to reference sequences in GenBank (≤ 81% nucleic acid identity, ≤79% amino acid identity).

### Community spatial structure of thaumarchaeal *accA* genes

**Figure 6:**
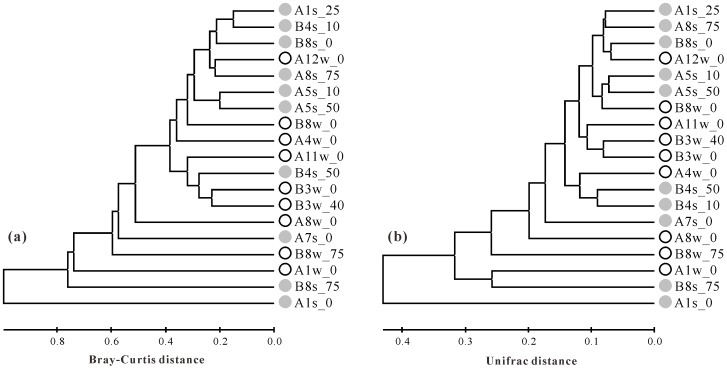
Clustering of the thaumarchaeal *accA* gene clone libraries based on the Bray-Curtis algorithm of OTU table (≤ 0.5% cutoff) (a) and the weighted UniFrac algorithm (b). Open circles (white) and close circles (grey) indicate samples obtained from autumn and summer, respectively. Scale bar indicates the Bray-Curtis (a) or the UniFrac distance (b).

**Figure 7:**
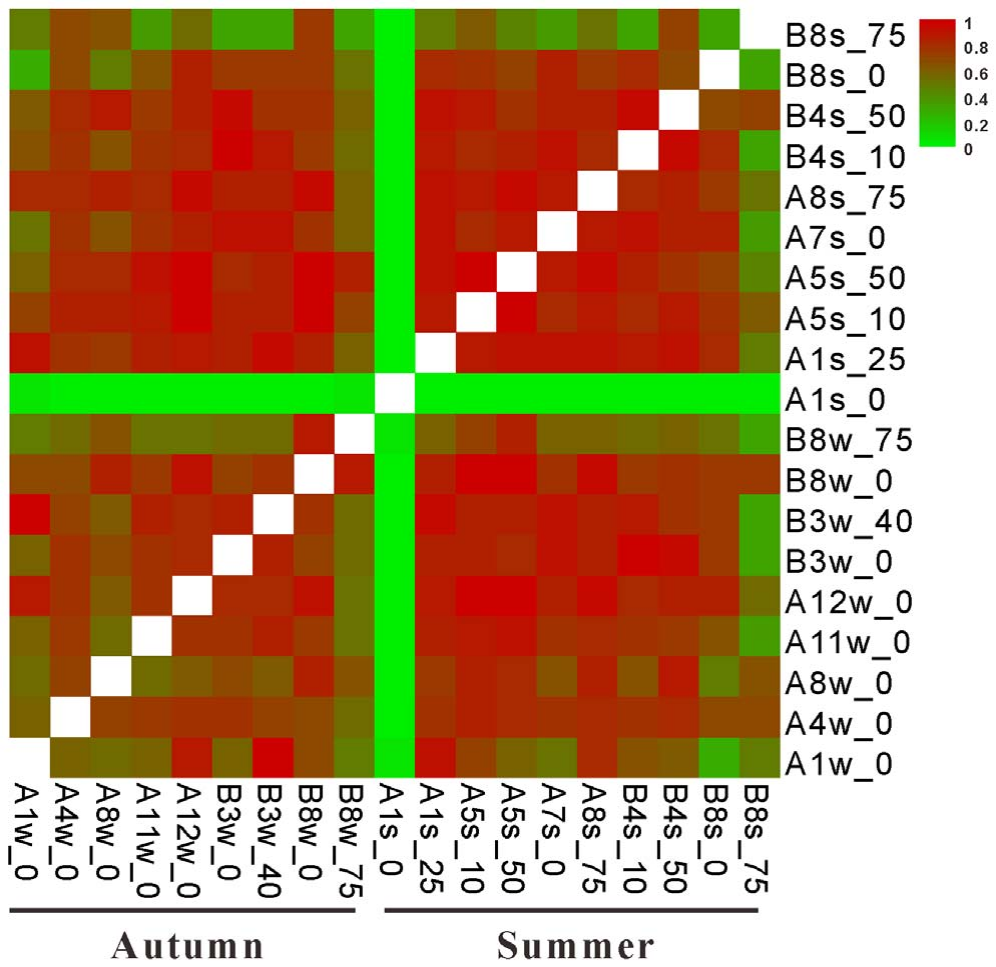
Proportion of shared OTUs based on abundance-based Sørensen-type (L_abd_) similarity between different thaumarchaeal *accA* clone libraries. OTUs clustered at a cutoff of 95% identity level.

Genetic differentiation among the thaumarchaeal *accA* clone libraries obtained from the ECS was assessed using both OTU-based cluster and weighted UniFrac cluster analysis (Fig. 6). Both cluster analyses demonstrated almost identical patterns (Mantel test, r = 0.95, *P*<0.001), and there was no clear environment clustering with regard to season (ANOSIM analysis, R = −0.07, *P*>0.05). Most of the clone libraries (13 out of 19) clustered together, sharing more than 60% Bray-Curtis or 85% UniFrac similarity (Fig. 6). Two continental shelf clone libraries (A7s_0 and A8w_0) were closer to the clone libraries mentioned above, whereas two surface samples from estuarine station A1 and two deep water samples from continental shelf station B8 shared lower similarity with other clone libraries (Fig. 6). This cluster pattern was also supported by analysis of the number of OTUs shared between any two clone libraries. For example, most clone libraries, either autumn or summer, exhibited significant compositional overlap (L_abd_≥0.5) except that A1s_0 shared almost no OTUs with other samples (L_abd_≤0.06), and A1w_0, B8w_75 and B8s_75 shared a relatively lower number of OTUs with several samples (L_abda_<0.5) (Fig. 7).

Statistical analysis demonstrated that hydrographic conditions might have had significant effects on the community structures of the autotrophic *Thaumarchaeota* (Table 2). Mantel tests indicated significant positive correlations between autotrophic thaumarchaeal communities and hydrographic parameters (temperature and salinity) (Table 2). Taken together, these results suggested that some ubiquitous lineages of autotrophic *Thaumarchaeota* were distributed in the ECS but that hydrographic factors shaped its community spatial structures.

**Table 2:** Mantel test for the thaumarchaeal *accA* community.

	Distance	Temp^a^	Sal^a^	Temp & Sal
Full community	0.10	**0.40** *	**0.51** *	0.57**
Surface community	0.09	0.15	**0.58** *	**0.45** *
Autumn community	0.18	0.29	0.79**	**0.61** *
Summer community	0.15	0.35	0.47	**0.56** *

a. Temp, Temperature; Sal, Salinity;

* . P<0.05; ** P<0.01.

## Discussion

The recently described AOA, responsible for the first and rate-limiting step of nitrification (ammonia oxidation), have emerged as an important microbial functional group in the global N cycle. Thaumarchaeal *amoA* genes are reported to be more abundant than those of their bacterial counterparts in diverse environments, especially in oligotrophic habitats [8], [10], [38]. Although field experiments or genome based studies demonstrate that AOA may live chemolithoautotrophically or mixotrophically [3], [18], [42], [43], limited information is available regarding the contributions of autotrophic *Thaumarchaeota* to the global carbon cycle, or environmental factors that control their distribution and abundance. To better understand the ecology of this novel primary producer, we investigated the abundance, distribution and community structure of autotrophic *Thaumarchaeota* in the shelf area of the ECS in two seasons (autumn and summer).

Our study indicated that the abundance of thaumarchaeal 16S rRNA and *accA* genes exhibited clear seasonal variation, being higher in late autumn (November) and lower in summer, although the abundance of the thaumarchaeal *accA* gene was quite low in the surface waters of the ECS shelf investigated (Figs 3 and 4). This was consistent with the fact that periodical blooms of *Thaumarchaeota* or AOA occur in other coastal oceans during late autumn-winter, including the North Sea [10], [44], Mediterranean Sea [45], Antarctic Ocean [46], [47] as well as Arctic coastal waters [48]. In addition, the strong relationship (positive correlation) between the dynamics of thaumarchaeal 16S rRNA and *accA* genes implied similar responses of the total and autotrophic *Thaumarchaeota* to environmental controls. Among possible planktonic *Thaumarchaeota* regulatory factors, temperature, nutrient levels, light availability and phytoplankton abundance and composition are considered as possible candidates [44], [48]–[50]. Previous studies propose that AOA may live chemolithotrophically since the 3-HP/4-HB pathway can be found in all published thaumarchaeal genomes, and ammonia oxidation of *Thaumarchaeota* is thought to provide the energy for CO_2_ fixation [18], [20]–[23], [42]. However, our qPCR results indicated that only 16.3% of planktonic *Thaumarchaeota* harbored the key gene for thaumarchaeal autotrophic pathways. This result was in accordance with our previous findings in the epipelagic waters of the ECS and SCS [7], [26], but appeared contrary to the metagenomic studies, which show a nearly 1∶1 ratio of thaumarchaeal *accA* to 16S rRNA gene in the surface water of the Sargasso Sea [12] and Gulf of Maine [51]. This incongruity might be explained by the following two reasons. On the one hand, some thaumarchaeal subpopulations in the coastal ECS might have lacked the *accA* gene or harbored alternative autotrophic CO_2_ fixation pathways. On the other hand, the thaumarchaeal *accA* primers used in the present study might have missed certain fraction of epipelagic *Thaumarchaeota* since those primers were primarily designed based on a few sequences (six and two sequences derived from metagenomic studies and thaumarchaeal strains, respectively) [7].

To obtain a more detailed understanding of the coverage and sensitivity of primers Cren529F and Cren981R for thaumarchaeal *accA* genes, we retrieved 22 full- or nearly full-length thaumarchaeal *accA* gene sequences from GenBank and CAMERA database (http://camera.calit2.net/) (as at 30 January 2013), and an in silico comparison of primer sets Cren529F/Cren981R to these sequences was performed (Table 3). The analysis revealed that the mismatches between thaumarchaeal *accA* gene primers used in this study and reference sequences were not uniformly distributed among the phylogenetic groups (Table 3). For instance, both forward and reverse *accA* primers had no mismatch with members of the deep cluster, whereas primers Cren529F and Cren981R had 1 ± 0.45 and 1.5 ± 0.34 mismatches per sequence, respectively, with the shallow cluster. Moreover, a relatively high frequency of mismatches between primer pair Cren529F/Cren981R and members of Group 1.1a-associated *Thaumarchaeota* was observed, especially for primer Cren981R (Table 3). Previous studies show that primer mismatches may result in a lower number of molecules being detected in qPCR assays [52], [53]. As a consequence, the abundance of the shallow cluster and Group 1.1a-associated group might be underestimated relative to the deep cluster of autotrophic *Thaumarchaeota* although our clone libraries were dominated by the sequences related to the shallow cluster. Further studies based on newly designed or modified *accA* primers are required to obtain a comprehensive view of the diversity and abundance of autotrophic *Thaumarchaeota*.

**Table 3:** Summary of thaumarchaeal *accA* gene mismatches to primer pair Cren529F/Cren981R.

Phylogenetic groups^a^	Sequence or strain name	Accession number	Cren529F (5'–3')	Cren981R (5'–3')
			GCW ATG ACW GAY TTT GTY RTA ATG	TGG WTK RYT TGC AAY TAT WCC
Shallow cluster	Sargasso Sea metagenome 1095460025585	AACY020784810	No mismatch	= = = = = = = = = = = = = = = A = = = = =
	Sargasso Sea metagenome 1096626735572	AACY020559552	No mismatch	= = = = = = = = = = = = = = = A = = = = =
	Sargasso Sea metagenome IBEA_CTG_2097946	AACY01016865	= = G = = = = = = = = = = = = = = A = = C = = =	= = = = = = = = = = = = = = = A = = = = =
	Sargasso Sea metagenome IBEA_CTG_2151838	AACY01042731	= = = = = = = = = = = = = = = = = = = = C = = =	= = = = = = = = = A = = = = = A = = G = =
	Sargasso Sea metagenome IBEA_CTG_2033382	AACY01063159	= = = = = = = = = = = = = = = = = = = = C = = =	= = = = = = = = = = = = = = = A = = = = =
	Sargasso Sea metagenome IBEA_CTG_UAAYO84TF	AACY01523534	= = = = = = = = = = = = = = = = = = = = T = = =	= = = = = = = = = = = = = = = A = C = = =
Deep cluster	HF4000_ANIW97P9 fosmid clone	EU016593	No mismatch	No mismatch
	HE4000_APKG6D3 fosmid clone	EU016643	No mismatch	No mismatch
	Marine metagenome HOTS_Contig54507	ABEF01054507	No mismatch	No mismatch
	Marine metagenome HOTS_Contig48812	ABEF01048812	No mismatch	No mismatch
Group 1.1a-associated	‘Gulf of Maine’ metagenome scf1108793271369	JH165397	= = = = = = = = = = = = = = = = = = = = T = = =	= = = = = = = = = G = = = = = A = = = = =
	‘Gulf of Maine’ metagenome scf1108793271546	JH165451	= = = = = = = = = = = = = = = = = = = = T = = =	= = = = = = = = = A = = T = = A = = = = =
	*Nitrosopumilus maritimus* SCM1	NZ_ABEO01000001	No mismatch	No mismatch
	*Nitrosopumilus salaria* BD31	NZ_AEXL01000070	= = = = = = = = = = = = = = = = = A = = T = = =	A = = = = = = = = A = = T = = G = = = = =
	*Nitrosopumilus koreensis* AR1	CP003842	= = = = = = = = = = = = = = = = = = = = C = = =	= = = = = = = = = = = = = = = A = = = = =
	*Nitrosopumilus* sp. SJ	AJVI01000006	= = = = = = = = = = = = = = = = = = = = C = = =	= = = = = = = = = = = = = = = A = = = = =
	*Nitrosopumilus* sp. AR	AJVJ01000016	= = = = = = = = = = = = = = = = = = = = C = = =	= = = = = = = = = = = = = = = A = = = = =
	*Nitrosopumilus sediminis* AR2	CP003843	= = = = = = = = C = = = = = = = = A = = T = = =	= = = = = = = = = = = = = = = A = = = = =
	*Nitrosoarchaeum limnia* BG20	AHJG01000246	= = = = = = = = = = = = = = = = = = = = T = = =	= = = = = = = = = A = = = = = G = = = = =
	*Nitrosoarchaeum limnia* SFB1	NZ_CM001158	= = = = = = = = = = = = = = = = = = = = T = = =	C = = = = = = = = A = = = = = G = = = = =
	*Nitrosoarchaeum koreensis* MY1	NZ_AFPU01000001	= = = = = = = = = = = = = = = = = = = = T = = =	= = = = = = = = = A = = = = = A = = = = =
Cenarchaeum	*Cenarchaeum symbiosum*	DP000238	= = C = = = = = G = = = = = = = = G = = = = = =	C = = C = = = = = G = = C = = = = = = = =
Group 1.1b	*Nitrososphaera gargensis* Ga9.2	CP002408	= = = = = = = = C = = = = = = = = G = = = = = =	C = = C = = = = = = = = G = = A = = G = =

Phylogenetic groups were identified based on thaumarchaeal *accA* genes.

The autotrophic thaumarchaeal diversity in the shelf water of the ECS (3–13 OTUs) revealed by *accA* gene sequences was comparable to previously studied open regions of the ECS (3–17 OTUs) [7], SCS (5–9 OTUs) [26] and Mediterranean Sea (4–6 OTUs) [24]. However, phylogenetic analysis indicated that the majority of thaumarchaeal *accA* gene sequences (86.3%) obtained from the coastal waters of the ECS, even from twilight zone waters (40–75 m) [54], were affiliated with the shallow cluster (Fig. 5), although the deep cluster was extensively retrieved below the euphotic zone in the open ECS (bathymetry >200 m) [26]. Vertical phylogenetic segregation of *Thaumarchaeota* in the ocean is observed in numerous studies based on analysis of 16S rRNA and different functional genes (*accA*, *amoA*, *nirK*, ureC and 4-*hcd*) of marine *Thaumarchaeota* [7], [25], [39], [55]. Several environmental parameters, such as light level, temperature and oxygen concentration, are considered as the critical factors in the development of the depth stratification phylogeny of marine planktonic *Thaumarchaeota* [56]. However, two recent studies argue that community structures of Group 1.1a are significantly influenced by nutrient status (ammonia concentrations) of the ocean rather than light gradients [57], [58]. These authors note that shallow and deep ecotypes of AOA dominate marine environments with medium and low ammonia concentrations, respectively. Considering the slightly higher concentration of ammonia (0.07–0.65 µM) present in the ECS shelf [59], it is possible that a predominance of shallow ecotypes of Group 1.1a was detected in the current study. This result agreed well with the observation that shallow Group 1.1a dominate throughout the water column down to 300m of the coastal Arctic [58], providing further evidence that ammonia supply rates might play a pivotal role in determining the community structures of the autotrophic *Thaumarchaeota*.

Although only a few *accA* OTUs (three OTUs, 66.5% of total sequences) had cosmopolitan distribution in the ECS shelf, a uniform seasonal distribution pattern was found for most OTUs (Fig. 5). This pattern was confirmed by cluster and ANOSIM analysis, which indicated that community structures of the autotrophic *Thaumarchaeota* did not differ markedly between autumn and summer. However, a Mantel test demonstrated that the community structures of the autotrophic *Thaumarchaeota* in the ECS shelf were closely tied to water mass properties, i.e. temperature and salinity either within a season or between seasons (Table 2). This observation agreed with those of previous studies, which show that planktonic archaeal communities may tend to be shaped by complex hydrographic conditions in the Arctic Ocean where different water masses meet [60], [61], although Massana et al. (2000) note that a few abundant OTUs of planktonic Archaea exhibited a ubiquitous distribution pattern in the world’s oceans [62].

The Changjiang is the third largest river in the world and contributes huge anthropogenic inputs of nutrient and organic matter to the ECS [63], [64]. Previous studies show that CDW brings terrestrial (allochthonous) source microorganisms to the Changjiang estuary and the adjacent ECS [30], [65]. Consequently, greater bacterial and archaeal diversity is observed in that area [30], [65], [66]. As expected, the highest diversity of autotrophic *Thaumarchaeota* was found in the surface water of estuarine station A1 in summer 2008 in our study (Table 1), where strong freshwater plumes were observed (Fig. 2d). Furthermore, most of the thaumarchaeal *accA* gene OTUs (69.2%) obtained from sample A1s_0 were exclusively related to Group 1.1b *Thaumarchaeota* and formed a unique monophylogenetic cluster (Fig. 5). Because this group was only observed in summer (when freshwater plumes became larger) but not in autumn (Fig. 2), it might have originated from terrestrial habitats. Although there is no universal genetic threshold for distinguishing autotrophic thaumarchaeal species (≤ 87% nucleic acid identity for thaumarchaeal *amoA* gene) [67], the high divergence between the Estuarine group and reference sequences (≤ 81% nucleic acid identity) indicated that a novel putative terrestrial cluster of autotrophic *Thaumarchaeota* was observed, highlighting the importance of freshwater runoff in the transport of terrestrial autotrophic *Thaumarchaeota* to marine environments.

In conclusion, the distribution, abundance and community structure of the autotrophic *Thaumarchaeota* were investigated for the first time along four transects in the ECS shelf during different seasons. The abundance of thaumarchaeal 16S rRNA and *accA* genes showed strong seasonal dynamics, whereas the community structures of autotrophic *Thaumarchaeota* might have been affected by the hydrographic conditions, i.e. temperature and salinity. Although caution is needed before establishing a direct link between *accA* gene abundance and autotrophic *Thaumarchaeota* activity, our results suggested that dark CO_2_ fixation by *Thaumarchaeota* might be more important in the late-autumn ECS ecosystem, which was in agreement with the high expressions of thaumarchaeal 3-HB/4-HP cycle-related genes in the Antarctic Peninsula coastal surface waters during the winter [49], [68].

## References

[1] Woese CR, Fox GE (1977) Phylogenetic structure of the prokaryotic domain: the primary kingdoms. Proc Natl Acad Sci U S A 74: 5088–5090. 270744 10.1073/pnas.74.11.5088 PMC432104

[2] DeLong EF (1998) Everything in moderation: Archaea as 'non-extremophiles'. Curr Opin Genet Dev 8: 649–654. 9914204 10.1016/s0959-437x(98)80032-4

[3] Herndl GJ, Reinthaler T, Teira E, van Aken H, Veth C, et al (2005) Contribution of Archaea to total prokaryotic production in the deep Atlantic Ocean. Appl Environ Microbiol 71: 2303–2309. 15870315 10.1128/AEM.71.5.2303-2309.2005 PMC1087563

[4] Karner MB, DeLong EF, Karl DM (2001) Archaeal dominance in the mesopelagic zone of the Pacific Ocean. Nature 409: 507–510. 11206545 10.1038/35054051

[5] Brochier-Armanet C, Boussau B, Gribaldo S, Forterre P (2008) Mesophilic Crenarchaeota: proposal for a third archaeal phylum, the Thaumarchaeota. Nat Rev Microbiol 6: 245–252. 18274537 10.1038/nrmicro1852

[6] Spang A, Hatzenpichler R, Brochier-Armanet C, Rattei T, Tischler P, et al (2010) Distinct gene set in two different lineages of ammonia-oxidizing archaea supports the phylum Thaumarchaeota. Trends Microbiol 18: 331–340. 20598889 10.1016/j.tim.2010.06.003

[7] Hu A, Jiao N, Zhang CL (2011) Community structure and function of planktonic Crenarchaeota: changes with depth in the South China Sea. Microb Ecol 62: 549–563. 21597940 10.1007/s00248-011-9866-z

[8] Leininger S, Urich T, Schloter M, Schwark L, Qi J, et al (2006) Archaea predominate among ammonia-oxidizing prokaryotes in soils. Nature 442: 806–809. 16915287 10.1038/nature04983

[9] Offre P, Nicol GW, Prosser JI (2010) Community profiling and quantification of putative autotrophic thaumarchaeal communities in environmental samples. Environ Microbiol Rep 3: 245–253. 23761257 10.1111/j.1758-2229.2010.00217.x

[10] Wuchter C, Abbas B, Coolen MJL, Herfort L, van Bleijswijk J, et al (2006) Archaeal nitrification in the ocean. Proc Natl Acad Sci U S A 103: 12317–12322. 16894176 10.1073/pnas.0600756103 PMC1533803

[11] Treusch AH, Leininger S, Kletzin A, Schuster SC, Klenk HP, et al (2005) Novel genes for nitrite reductase and Amo-related proteins indicate a role of uncultivated mesophilic crenarchaeota in nitrogen cycling. Environ Microbiol 7: 1985–1995. 16309395 10.1111/j.1462-2920.2005.00906.x

[12] Venter JC, Remington K, Heidelberg JF, Halpern AL, Rusch D, et al (2004) Environmental genome shotgun sequencing of the Sargasso Sea. Science 304: 66–74. 15001713 10.1126/science.1093857

[13] de la TorreJR, Walker CB, Ingalls AE, Konneke M, Stahl DA (2008) Cultivation of a thermophilic ammonia oxidizing archaeon synthesizing crenarchaeol. Environ Microbiol 10: 810–818. 18205821 10.1111/j.1462-2920.2007.01506.x

[14] Hatzenpichler R, Lebedeva EV, Spieck E, Stoecker K, Richter A, et al (2008) A moderately thermophilic ammonia-oxidizing crenarchaeote from a hot spring. Proc Natl Acad Sci U S A 105: 2134–2139. 18250313 10.1073/pnas.0708857105 PMC2538889

[15] Könneke M, Bernhard AE, de la TorreJR, Walker CB, Waterbury JB, et al (2005) Isolation of an autotrophic ammonia-oxidizing marine archaeon. Nature 437: 543–546. 16177789 10.1038/nature03911

[16] Francis CA, Beman JM, Kuypers MMM (2007) New processes and players in the nitrogen cycle: the microbial ecology of anaerobic and archaeal ammonia oxidation. ISME J 1: 19–27. 18043610 10.1038/ismej.2007.8

[17] Hallam SJ, Mincer TJ, Schleper C, Preston CM, Roberts K, et al (2006) Pathways of carbon assimilation and ammonia oxidation suggested by environmental genomic analyses of marine Crenarchaeota. PLoS Biol 4: e95. 16533068 10.1371/journal.pbio.0040095 PMC1403158

[18] Walker CB, de la TorreJR, Klotz MG, Urakawa H, Pinel N, et al (2010) *Nitrosopumilus maritimus* genome reveals unique mechanisms for nitrification and autotrophy in globally distributed marine crenarchaea. Proc Natl Acad Sci U S A 107: 8818–8823. 20421470 10.1073/pnas.0913533107 PMC2889351

[19] Berg IA, Kockelkorn D, Buckel W, Fuchs G (2007) A 3-hydroxypropionate/4-hydroxybutyrate autotrophic carbon dioxide assimilation pathway in archaea. Science 318: 1782–1786. 18079405 10.1126/science.1149976

[20] Blainey PC, Mosier AC, Potanina A, Francis CA, Quake SR (2011) Genome of a low-salinity ammonia-oxidizing archaeon determined by single-cell and metagenomic analysis. PLoS One 6: e16626. 21364937 10.1371/journal.pone.0016626 PMC3043068

[21] Kim BK, Jung MY, Yu DS, Park SJ, Oh TK, et al (2011) Genome sequence of an ammonia-oxidizing soil archaeon,“Candidatus Nitrosoarchaeum koreensis” MY1. J Bacteriol 193: 5539–5540. 21914867 10.1128/JB.05717-11 PMC3187385

[22] Mosier AC, Allen EE, Kim M, Ferriera S, Francis CA (2012) Genome sequence of “Candidatus Nitrosoarchaeum limnia” BG20, a low-salinity ammonia-oxidizing archaeon from the San Francisco Bay Estuary. J Bacteriol 194: 2119–2120. 22461554 10.1128/JB.00007-12 PMC3318456

[23] Mosier AC, Allen EE, Kim M, Ferriera S, Francis CA (2012) Genome sequence of “Candidatus Nitrosopumilus salaria” BD31, an ammonia-oxidizing archaeon from the San Francisco Bay Estuary. J Bacteriol 194: 2121–2122. 22461555 10.1128/JB.00013-12 PMC3318490

[24] Yakimov MM, Conoa VL, Denaroa R (2009) A first insight into the occurrence and expression of functional *amoA* and *accA* genes of autotrophic and ammonia-oxidizing bathypelagic Crenarchaeota of Tyrrhenian Sea. Deep-Sea Res II 56: 748–754.

[25] Yakimov MM, La Cono V, Smedile F, DeLuca TH, Juarez S, et al (2011) Contribution of crenarchaeal autotrophic ammonia oxidizers to the dark primary production in Tyrrhenian deep waters (Central Mediterranean Sea). ISME J 5: 945–961. 21209665 10.1038/ismej.2010.197 PMC3131861

[26] Hu A, Jiao N, Zhang R, Yang Z (2011) Niche partitioning of marine group I Crenarchaeota in the euphotic and upper mesopelagic zones of the East China Sea. Appl Environ Microbiol 77: 7469–7478. 21873485 10.1128/AEM.00294-11 PMC3209141

[27] Jiao NZ, Yang YH, Hong N, Ma Y, Harada S, et al (2005) Dynamics of autotrophic picoplankton and heterotrophic bacteria in the East China Sea. Cont Shelf Res 25: 1265–1279.

[28] Zhang Y, Jiao N (2007) Dynamics of aerobic anoxygenic phototrophic bacteria in the East China Sea. FEMS Microbiol Ecol 61: 459–469. 17617220 10.1111/j.1574-6941.2007.00355.x

[29] Zeng YH, Li HY, Jiao NZ (2007) Phylogenetic diversity of planktonic archaea in the estuarine region of East China Sea. Microbiol Res 162: 26–36. 16914298 10.1016/j.micres.2006.03.007

[30] Dang HY, Zhang XX, Sun J, Li TG, Zhang ZN, et al (2008) Diversity and spatial distribution of sediment ammonia-oxidizing crenarchaeota in response to estuarine and environmental gradients in the Changjiang Estuary and East China Sea. Microbiology 154: 2084–2095. 18599836 10.1099/mic.0.2007/013581-0

[31] Mincer TJ, Church MJ, Taylor LT, Preston C, Karl DM, et al (2007) Quantitative distribution of presumptive archaeal and bacterial nitrifiers in Monterey Bay and the North Pacific Subtropical Gyre. Environ Microbiol 9: 1162–1175. 17472632 10.1111/j.1462-2920.2007.01239.x

[32] Ludwig W, Strunk O, Westram R, Richter L, Meier H, et al (2004) ARB: a software environment for sequence data. Nucleic Acids Res 32: 1363–1371. 14985472 10.1093/nar/gkh293 PMC390282

[33] Stamatakis A, Hoover P, Rougemont J (2008) A rapid bootstrap algorithm for the RAxML Web servers. Syst Biol 57: 758–771. 18853362 10.1080/10635150802429642

[34] Schloss PD, Westcott SL, Ryabin T, Hall JR, Hartmann M, et al (2009) Introducing mothur: open-source, platform-independent, community-supported software for describing and comparing microbial communities. Appl Environ Microbiol 75: 7537–7541. 19801464 10.1128/AEM.01541-09 PMC2786419

[35] Lozupone C, Hamady M, Knight R (2006) UniFrac - An online tool for comparing microbial community diversity in a phylogenetic context. BMC Bioinformatics 7: 371. 16893466 10.1186/1471-2105-7-371 PMC1564154

[36] Hammer Ø, Harper DAT, Ryan PD (2001) PAST: paleontological statistics software package for education and data analysis. Palaeontol Electron. pp. 9.

[37] Jiao NZ, Yang YH, Koshikawa H, Watanabe M (2002) Influence of hydrographic conditions on picoplankton distribution in the East China Sea. Aquat Microb Ecol 30: 37–48.

[38] Beman JM, Popp BN, Francis CA (2008) Molecular and biogeochemical evidence for ammonia oxidation by marine Crenarchaeota in the Gulf of California. ISME J 2: 429–441. 18200070 10.1038/ismej.2007.118

[39] Santoro AE, Casciotti KL, Francis CA (2010) Activity, abundance and diversity of nitrifying archaea and bacteria in the central California Current. Environ Microbiol 12: 1989–2006. 20345944 10.1111/j.1462-2920.2010.02205.x

[40] DeLong EF, Preston CM, Mincer T, Rich V, Hallam SJ, et al (2006) Community genomics among stratified microbial assemblages in the ocean's interior. Science 311: 496–503. 16439655 10.1126/science.1120250

[41] Park BJ, Park SJ, Yoon DN, Schouten S, Damste JSS, et al (2010) Cultivation of autotrophic ammonia-oxidizing archaea from marine sediments in coculture with sulfur-oxidizing bacteria. Appl Environ Microbiol 76: 7575–7587. 20870784 10.1128/AEM.01478-10 PMC2976178

[42] Hallam SJ, Konstantinidis KT, Putnam N, Schleper C, Watanabe Y, et al (2006) Genomic analysis of the uncultivated marine crenarchaeote *Cenarchaeum symbiosum* . Proc Natl Acad Sci U S A 103: 18296–18301. 17114289 10.1073/pnas.0608549103 PMC1643844

[43] Ouverney CC, Fuhrman JA (2000) Marine planktonic Archaea take up amino acids. Appl Environ Microbiol 66: 4829–4833. 11055931 10.1128/aem.66.11.4829-4833.2000 PMC92387

[44] Pitcher A, Wuchter C, Siedenberg K, Schouten S, Sinninghe Damsté JS (2011) Crenarchaeol tracks winter blooms of ammonia-oxidizing Thaumarchaeota in the coastal North Sea. Limnol Oceanogr 56: 2308–2318.

[45] Galand PE, Gutiérrez-Provecho C, Massana R, Gasol JM, Casamayor EO (2010) Inter-annual recurrence of archaeal assemblages in the coastal NW Mediterranean Sea (Blanes Bay Microbial Observatory). Limnol Oceanogr 55: 2117–2125.

[46] Church MJ, DeLong EF, Ducklow HW, Karner MB, Preston CM, et al (2003) Abundance and distribution of planktonic Archaea and Bacteria in the waters west of the Antarctic Peninsula. Limnol Oceanogr 48: 1893–1902.

[47] Murray AE, Preston CM, Massana R, Taylor LT, Blakis A, et al (1998) Seasonal and spatial variability of bacterial and archaeal assemblages in the coastal waters near Anvers Island, Antarctica. Appl Environ Microbiol 64: 2585–2595. 9647834 10.1128/aem.64.7.2585-2595.1998 PMC106430

[48] Christman GD, Cottrell MT, Popp BN, Gier E, Kirchman DL (2011) Abundance, diversity, and activity of ammonia-oxidizing prokaryotes in the coastal arctic ocean in summer and winter. Appl Environ Microbiol 77: 2026–2034. 21239542 10.1128/AEM.01907-10 PMC3067342

[49] Grzymski JJ, Riesenfeld CS, Williams TJ, Dussaq AM, Ducklow H, et al (2012) A metagenomic assessment of winter and summer bacterioplankton from Antarctica Peninsula coastal surface waters. ISME J 6: 1901–1915. 22534611 10.1038/ismej.2012.31 PMC3446801

[50] Herfort L, Schouten S, Abbas B, Veldhuis MJW, Coolen MJL, et al (2007) Variations in spatial and temporal distribution of Archaea in the North Sea in relation to environmental variables. FEMS Microbiol Ecol 62: 242–257. 17991018 10.1111/j.1574-6941.2007.00397.x

[51] Tully BJ, Nelson WC, Heidelberg JF (2012) Metagenomic analysis of a complex marine planktonic thaumarchaeal community from the Gulf of Maine. Environ Microbiol 14: 254–267. 22050608 10.1111/j.1462-2920.2011.02628.x

[52] Boyle B, Dallaire N, MacKay J (2009) Evaluation of the impact of single nucleotide polymorphisms and primer mismatches on quantitative PCR. BMC Biotechnol 9: 75. 19715565 10.1186/1472-6750-9-75 PMC2741440

[53] Sipos R, Székely AJ, Palatinszky M, Révész S, Márialigeti K, et al (2007) Effect of primer mismatch, annealing temperature and PCR cycle number on 16S rRNA gene-targetting bacterial community analysis. FEMS Microbiol Ecol 60: 341–350. 17343679 10.1111/j.1574-6941.2007.00283.x

[54] Shang S, Lee Z, Wei G (2011) Characterization of MODIS-derived euphotic zone depth: Results for the China Sea. Remote Sens Environ 115: 180–186.

[55] Lund MB, Smith JM, Francis CA (2012) Diversity, abundance and expression of nitrite reductase (*nirK*)-like genes in marine thaumarchaea. ISME J 6: 1966–1977. 22592819 10.1038/ismej.2012.40 PMC3446794

[56] Biller SJ, Mosier AC, Wells GF, Francis CA (2012) Global biodiversity of aquatic ammonia-oxidizing archaea is partitioned by habitat. Front Microbiol 3: 252. 22826704 10.3389/fmicb.2012.00252 PMC3399221

[57] Molina V, Belmar L, Ulloa O (2010) High diversity of ammonia-oxidizing archaea in permanent and seasonal oxygen-deficient waters of the eastern South Pacific. Environ Microbiol 12: 2450–2465. 20406296 10.1111/j.1462-2920.2010.02218.x

[58] Sintes E, Bergauer K, De Corte D, Yokokawa T, Herndl GJ (2012) Archaeal *amoA* gene diversity points to distinct biogeography of ammonia-oxidizing Crenarchaeota in the ocean. Environ Microbiol: doi: 10.1111/j.1462-2920.2012.02801.x. 10.1111/j.1462-2920.2012.02801.x PMC3712475 22690844

[59] Zhang J, Liu S, Ren J, Wu Y, Zhang G (2007) Nutrient gradients from the eutrophic Changjiang (Yangtze River) Estuary to the oligotrophic Kuroshio waters and re-evaluation of budgets for the East China Sea Shelf. Prog Oceanogr 74: 449–478.

[60] Galand PE, Casamayor EO, Kirchman DL, Potvin M, Lovejoy C (2009) Unique archaeal assemblages in the Arctic Ocean unveiled by massively parallel tag sequencing. ISME J 3: 860–869. 19322244 10.1038/ismej.2009.23

[61] Galand PE, Lovejoy C, Hamilton AK, Ingram RG, Pedneault E, et al (2009) Archaeal diversity and a gene for ammonia oxidation are coupled to oceanic circulation. Environ Microbiol 11: 971–980. 19077007 10.1111/j.1462-2920.2008.01822.x

[62] Massana R, DeLong EF, Pedros-Alio C (2000) A few cosmopolitan phylotypes dominate planktonic archaeal assemblages in widely different oceanic provinces. Appl Environ Microbiol 66: 1777–1787. 10788339 10.1128/aem.66.5.1777-1787.2000 PMC101412

[63] Gao XL, Song JM (2006) Main geochemical characteristics and key biogeochemical carbon processes in the East China Sea. J Coast Res 22: 1330–1339.

[64] Yan WJ, Mayorga E, Li XY, Seitzinger SP, Bouwman AF (2010) Increasing anthropogenic nitrogen inputs and riverine DIN exports from the Changjiang River basin under changing human pressures. Global Biogeochem Cy 24: doi: 10.1029/2009GB003575.

[65] Feng BW, Li XR, Wang JH, Hu ZY, Meng H, et al (2009) Bacterial diversity of water and sediment in the Changjiang estuary and coastal area of the East China Sea. FEMS Microbiol Ecol 70: 236–248. 10.1111/j.1574-6941.2009.00772.x 19780829

[66] Liu M, Xiao T, Wu Y, Zhou F, Zhang W (2011) Temporal distribution of the archaeal community in the Changjiang Estuary hypoxia area and the adjacent East China Sea as determined by denaturing gradient gel electrophoresis and multivariate analysis. Can J Microbiol 57: 504–513. 21635218 10.1139/w11-037

[67] Pester M, Rattei T, Flechl S, Gröngröft A, Richter A, et al (2012) *amoA*-based consensus phylogeny of ammonia-oxidizing archaea and deep sequencing of *amoA* genes from soils of four different geographic regions. Environ Microbiol 14: 525–539. 22141924 10.1111/j.1462-2920.2011.02666.x PMC3328746

[68] Williams TJ, Long E, Evans F, DeMaere MZ, Lauro FM, et al (2012) A metaproteomic assessment of winter and summer bacterioplankton from Antarctic Peninsula coastal surface waters. ISME J 6: 1883–1900. 22534610 10.1038/ismej.2012.28 PMC3446797

